# Physical activity among hospitalised older people: insights from upper and lower limb accelerometry

**DOI:** 10.1007/s40520-018-0930-0

**Published:** 2018-03-14

**Authors:** S. E. R. Lim, R. Dodds, D. Bacon, A. A. Sayer, H. C. Roberts

**Affiliations:** 1 Academic Geriatric Medicine, University of Southampton, Southampton, UK; 2 NIHR CLAHRC Wessex, University of Southampton, Southampton, UK; 3 University Hospital Southampton NHS FT, Southampton, UK; 4 AGE Research Group, Institute of Neuroscience, Newcastle University, Newcastle upon Tyne, UK; 5 NIHR Newcastle Biomedical Research Centre, Newcastle upon Tyne Hospitals NHS Foundation Trust and Newcastle University, Newcastle upon Tyne, UK

**Keywords:** Older people, Hospital, Physical activity, Accelerometer

## Abstract

**Background:**

Few studies have explored the activity levels of hospitalised older people and the intra-daily activity patterns in this group have not been described.

**Aims:**

To describe the quantity and daily pattern of physical activity among hospitalised older people using two accelerometers: the ankle-worn StepWatch Activity Monitor (SAM), and the wrist-worn GENEActiv.

**Methods:**

This cross-sectional observational study was conducted on the acute medical wards for older people in one UK hospital. Inclusion criteria: participants aged ≥ 70 years, and able to mobilise prior to admission. Participants wore both devices for up to seven consecutive days, or until hospital discharge, whichever was sooner. Intra-daily activity levels were analysed hourly over each 24 h period.

**Results:**

38 participants (mean age 87.8 years, SD 4.8) had their activity levels measured using both devices. The SAM median daily step count was 600 (IQR 240–1427). Intra-daily activity analysis showed two peak periods of ambulatory activity between 9 am–11 am and 6 pm–7 pm. With physical activity defined as ≥ 12 milli-g (GENEActiv), the median time spent above this cut-off point was 4.2 h. 62% of this activity time was only sustained for 1–5 min. Acceptability of both devices was high overall, but the wrist-worn device (96%) was more acceptable to patients than the ankle-worn device (83%).

**Conclusion:**

Activity levels of these hospitalised older people were very low. Most physical activity was sustained over short periods. The intra-daily pattern of activity is an interesting finding which can help clinicians implement time-specific interventions to address the important issue of sedentary behaviour.

## Background

Deconditioning can be defined as the physiologic changes occurring with prolonged bed rest or other inactivity [1]. There is increasing recognition that deconditioning associated with physical inactivity during hospitalisation can result in a range of adverse effects for hospitalised older people, including increased frailty [2], functional decline [3] and development of disability in activities of daily living [4]. There is a growing interest in interventions promoting increased physical activity among older people in hospital [5, 6].

A recent systematic review [7] has highlighted how a range of different physical activity measures have been used in this patient group, including step count [8, 9], posture identification [10–12], and physical activity energy expenditure [13]. Studies conducted in the US suggest that hospitalised older people are inactive, with low daily step counts (478–846 steps) [14] and with little time spent in an upright position per day (43 min walking or standing) [10].

However, we are not aware of any studies which have described the daily pattern of activity levels of hospitalised older people in detail. Such information could be beneficial in the future when deciding the optimum time of day and duration of physical activity interventions. In addition, whilst many existing studies have focussed on step count or time spent standing, they have not captured activities in the seated position using accelerometry. This is an important area to address because amongst the less mobile, such activities may be the best target for intervention. The aim of this study was, therefore, to describe in detail the quantity and daily pattern of physical activity among hospitalised older people using simultaneous ankle- and wrist-worn accelerometry.

## Methods

### Study design and population

The data for this study come from the baseline phase of SoMoVe, a study examining the feasibility of implementing a volunteer-led physical activity intervention on acute medical wards for older people (ClinicalTrials.gov no: NCT02594527). This observational cross-sectional phase of the study was conducted between February and July 2016 before volunteers were trained. Patients admitted to the acute medical wards for older people in one hospital were invited to participate. Inclusion criteria included patients aged ≥ 70 years who were mobile prior to admission and able to provide valid written consent. Patients isolated for infection control reasons and those receiving end-of-life care were excluded. Patients were identified through discussions with the nurse in-charge on the study wards and by reviewing medical notes to determine if the inclusion criteria were met. Patients who were eligible to participate were approached and details about the study were provided including the patient information sheet. Written consent was obtained from patients who wished to participate. This study was approved the South East Coast—Surrey Research Ethics Committee.

### Measure of physical activity

Physical activity levels were measured using two accelerometers, the StepWatch Activity Monitor (SAM) (Modus health, Washington, US) and GENEActiv (Activinsights, Kimbolton, UK). Participants wore both devices for a maximum of 7 days or until hospital discharge, whichever was sooner. A minimum of 24 h recording from both devices was required for data analysis.

The SAM is an ankle-worn dual-axis accelerometer. Its primary output is stride count per minute, with each stride equivalent to two steps. It has been used in previous studies measuring step counts of hospitalised older people [14, 15] and has been reported to be accurate at slower gait speeds, down to 0.45 m/s [16]. The GENEActiv is a wrist-worn tri-axial accelerometer [17]. It records acceleration in three planes with a frequency of 100 Hz. Its output is summarised in the form of a signal magnitude vector of the three planes with the acceleration due to gravity subtracted (unit milli-g). The device contains a temperature sensor to allow detection of periods of non-wear.

### Covariates

Several covariates were recorded including Barthel Index [18], gait speed [19], grip strength [20], Charlson Comorbidity Index [21] and number of medications. The Barthel Index is a physical function measure which assesses patients’ functional abilities in activities of daily living: transfers, walking, stairs, toilet use, dressing, feeding, bladder control, bowel control, grooming and bathing, to give a maximum total score of 100. Gait speed was measured by recording the time taken for each participant to walk 4 m at a comfortable pace, using a stopwatch. Hand grip strength was measured using the Jamar dynamometer. Participants were seated either on the chair or upright in bed, with shoulders adducted and neutrally rotated, elbow flexed at 90° and wrist neutrally rotated. Grip strength was then tested on both hands twice and the highest of the four scores was recorded as the final score for maximum grip strength. A review of participants’ medical notes was conducted to collect data regarding comorbidities and number of medications.

### Device acceptability

A questionnaire was designed to explore the views of the participants regarding the comfort and ease of wearing the devices over the period of the recording and whether the devices interfered with any aspect of their daily activities including personal hygiene, washing, toileting, mobility and sleep. Upon removal of the devices, an interviewer went through the questionnaire with each participant and completed the questionnaire. Participants who were discharged out of hours did not complete the questionnaires.

### Statistical analyses

The SAM data were analysed using the software provided with the device; for the GeneActiv data we used the GGIR library [22] for the statistical package, R [23]. We performed all subsequent analyses using STATA version 14 [24]. We restricted each recording to the period when data were available from both devices and excluded hours with only partial data at either end of recordings.

The mean step count and the mean acceleration per hour of each day for each participant were analysed. Each minute of recording from each device was classified as a binary output (active or inactive) to make the output from the two devices comparable. We were not aware of existing cut-points for activity in this population, which is characterised by very low activity levels as previously described [10]. Each minute of the SAM recordings was classified as active where participants took four or more steps (two or more strides). Receiver operating characteristic curve analysis was used to determine an equivalent cut-point for the GENEActiv recordings. The analysis showed that a 1-min mean acceleration cut-point of ≥ 12 milli-g produced an area under the curve value of 0.822, with sensitivity and specificity of 82% for detecting if participants had taken four or more steps in the same minute. Activity periods were classified into bouts of 1–5 min, 6–10 min or greater than 10 min duration.

Mixed effects logistic regression was conducted to investigate the associations between hour of the day and the likelihood of activity from the above binary variables, whilst taking account of clustering of data at the level of each participant. Differences in activity by day of recording were investigated. In both logistic regression analyses, evidence of interaction by gender was tested.

## Results

67 inpatients met the inclusion and exclusion criteria and 50 participants consented to participate in the study. 38 participants (18 men and 20 women, mean ages 88.3 and 87.5 years, respectively) had at least a 24-h recording from both devices. The characteristics of participants are shown in Table 1. The median Barthel Index score was similar for men (80) and women (71). The median gait speed for both men and women was 0.47 m/s. The mean grip strength for men and women was 22.8 and 13 kg, respectively. The illnesses that commonly precipitated hospital admission included: pneumonia (18%), urinary sepsis (18%), musculoskeletal problems (18%) and heart failure (13%).

**Table 1:** Participants’ characteristics and physical activity levels

Characteristic	Men (*n* = 18)	Women (*n* = 20)
Age (years)^a^	88.3 (5.1)	87.5 (4.5)
Barthel Index^b^	80 (72, 92)	71 (45, 88)
Gait speed^b^ (*n* = 18)	0.47 (0.31, 0.77)	0.47 (0.17, 0.67)
Grip strength^a^ (*n* = 34)	22.8 (7.9)	13 (11, 17)
Charlson’s Comorbidity Index^b^	7 (6, 8)	7 (5, 9)
Number of medications^b^	7 (4, 11)	9 (6, 12)
Mini-mental state examination^b^	27 (23.5, 27)	22 (20, 28)
Diagnosis		
Pneumonia	4 (22%)	3 (15%)
Urinary sepsis	4 (22%)	3 (15%)
Heart failure	2 (11%)	3 (15%)
Musculoskeletal problems	3 (17%)	4 (20%)
Neurological problems	1 (5.5%)	2 (10%)
Other respiratory illnesses	3 (17%)	1 (5%)
Other source of sepsis	0	3 (15%)
Electrolyte imbalance	0	1 (5%)
No acute medical illness	1 (5.5%)	0
Recording length (days)^a^	4.8 (2.1)	3.8 (1.9)
Steps (SAM)		
Steps per day^b^	834 (316, 2161)	404 (211, 979)
Total minutes per day with ≥ 4 steps^b^	54 (24, 104)	32 (19, 54)
Minutes spent in sustained ambulation with ≥ 4 steps		
1–5 min^b^	46 (15, 77)	30 (19, 49)
6–10 min^b^	5 (0, 18)	1 (0, 4)
10+ min^b^	1 (0, 7)	0 (0,0)
Wrist acceleration (GeneActiv)		
Mean acceleration per minute (milli-g)^a^	9.9 (3.0)	7.8 (2.5)
Total minutes per day with acceleration ≥ 12 milli-g*^b^	315 (223, 377)	236 (165, 289)
Minutes spent in different bout lengths with acceleration ≥ 12milli-g		
1–5 min^b^	161 (125, 193)	139 (108, 176)
6–10 min^b^	53 (34, 68)	39 (20, 58)
10+ min^b^	61 (46, 158)	29 (19, 50)

*SAM* stepwatch activity monitor

^a^Mean (SD)

^b^Median (IQR)

*This cut-off point was chosen as being most comparable to taking four or more steps in a given minute

### Average step count and wrist acceleration across the day

**Fig. 1:**
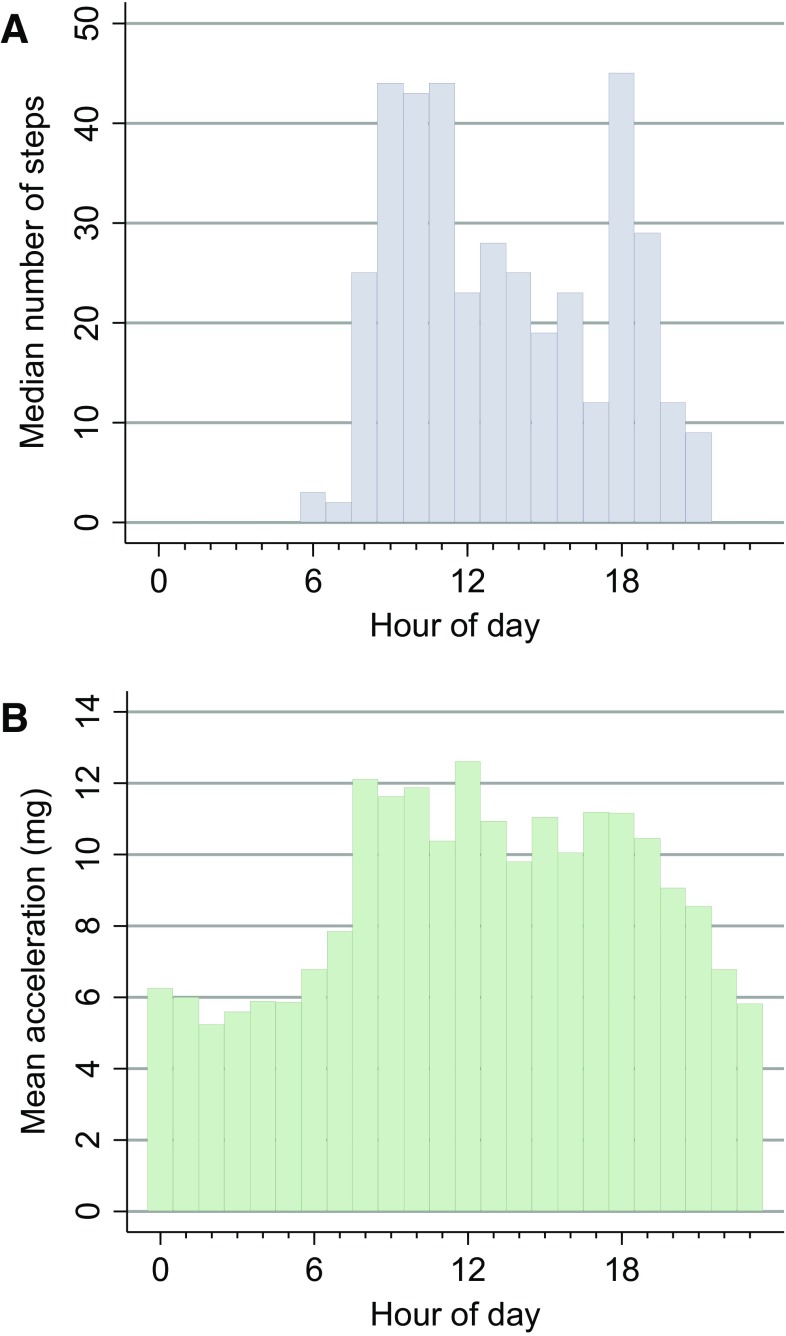
Step count and wrist acceleration by hour of day. Values shown are for all 38 participants. **a** Median number of steps per hour from the Stepwatch device. **b** Mean acceleration (milli-g) per hour from the GeneActiv wrist-worn device

The step count in the sample was positively skewed, with median 600 (IQR 240, 1427) steps per day and a trend towards a higher step count in men (Table 1). There were two peak periods of median step count throughout the day: 9–11 am and 6–7 pm (Fig. 1a).

The measured acceleration at the wrist was low with a mean of 8.8 milli-g per minute, with higher values in men than women (9.9 and 7.8 milli-g, respectively, *P* = 0.02). The mean values for acceleration per hour (Fig. 1b) suggested a general increase in activity during the daytime compared to the night.

### Number of minutes spent above cut-points across the day

Participants spent a median 40 min (IQR 20, 73) per day taking four steps or more per minute and these were typically in bouts of 5 min or less. By comparison, participants spent longer at or above the wrist acceleration cut-point of 12 milli-g: a median of 4 h and 19 min (IQR 3 h 10 min, 5 h 36 min) per day. Most of this activity was again in bouts of 1–5 min duration. Men tended to undertake a greater duration of activity than women, as shown in Table 1.

**Fig. 2:**
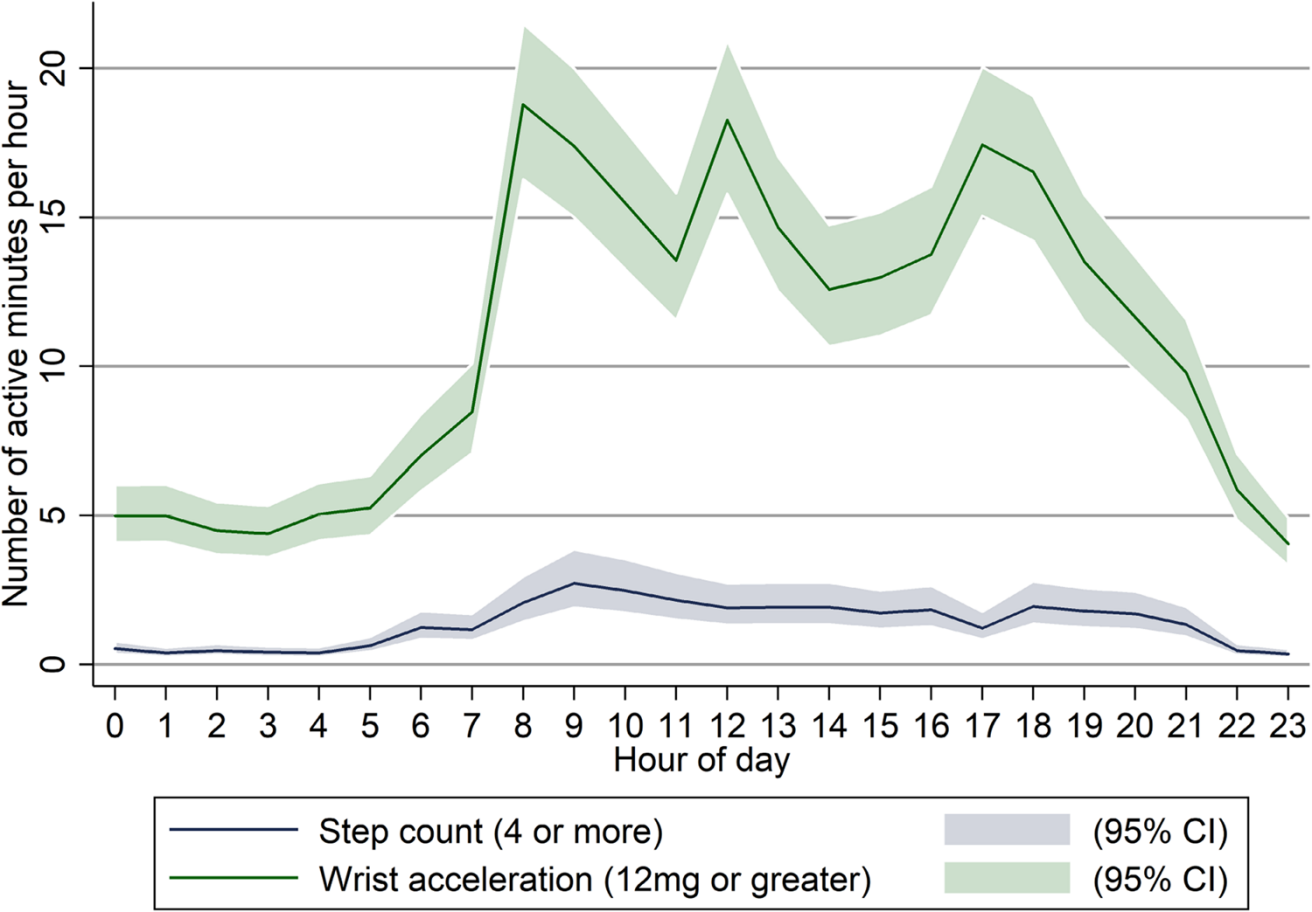
Number of minutes of activity at or above cut-points for step count and wrist acceleration, shown by hour of day. This graph shows the findings from the mixed effects logistic regression models (with the predicted probabilities multiplied by 60 to give number of minutes per hour), using the binary variables for step count and wrist acceleration for each minute as outcomes

The findings from the models for the likelihood of activity by time of day are shown in Fig. 2. They revealed low levels (1 or 2 min/h) of step activity between 6 am and 9 pm. By contrast, we saw sustained activity at the wrist over the same period, with peaks of approximately 18 min/h at 9 am, 12 pm and 5 pm. We did not find differences in activity by day over up to 5 successive days of recording.

### Device acceptability

23/38 (61%) participants (14 men) completed the questionnaire upon removal of the devices. Four participants (17%) reported that the SAM device was uncomfortable to wear overnight although it was well-tolerated during the day. Only one participant (4.3%) reported that the GENEActiv was uncomfortable to wear on the wrist; the participant in question had never regularly worn a watch.

## Discussion

### Summary of findings

Using simultaneous ankle- and wrist-worn physical activity devices, we have described the physical activity levels and patterns of 38 acute medical inpatients aged ≥ 70 years. Participants in this study appeared to be physically frail and at high risk of sarcopenia as suggested by a low median gait speed (0.47 m/s) and low median grip strength among men (22.8 kg) and women (13 kg). The European Working Group on Sarcopenia in Older People (EWGSOP) cut-off points for identifying sarcopenia are 0.8 m/s for gait speed [25] and < 32 kg for men and < 22 kg for women for grip strength [26].

We found overall low activity levels with both devices, with activity typically occurring in bouts of 5 min or shorter duration. We saw different patterns of activity from the two devices, with peaks in step count per hour between 9–11 am and 6–7 pm, compared to peaks in wrist acceleration at 9 am, 12 pm and 5 pm, which corresponds to patient meal times.

### Comparison with other studies

We are not aware of existing studies looking at ambulatory activity of older medical inpatients in the UK. However, the low median daily step count of 600 steps reported in this study appears to be consistent with findings from research in other countries. McCullagh et al. examined the mobility levels of 150 medical inpatients (mean age 77.5, SD 7.4) in a hospital in Ireland and reported a median daily step count ranging from 299 to 661 steps per day [27]. Studies in the US report a similar range of daily step count. Ostir et al [14] reported a median of 478 steps among patients aged > 65 years in the first 24 h of hospitalisation and 846 steps in the last 24 h of hospitalisation. Fisher et al [15] examined the step count of 162 patients who were admitted to Acute Care of the Elderly unit (mean age of 77.4 years) and reported a mean daily step count of 662.

### Interpretation of findings

The intra-daily variation in physical activity levels is a novel finding. Peak step counts occurred between the hours of 9–11 am and 6–7 pm. The increase in step count in the morning could be explained by higher levels of therapy input during this period as well as personal care. Step count then declined steadily from lunch time onwards and was at the lowest towards the end of visiting hours. The increase in step count was noted again later in the evening between 6 and 7 pm, which could be due to patients preparing themselves for bedtime and may include trips to the bathroom for personal care. An interesting finding of this study was the low step count during visiting hours. An observed culture on the wards was that visitors tended to gather around patients’ bedside rather than encouraging them to be more active and taking them out for walks. A cultural change may be needed to empower family members and friends to encourage patients to be more active in hospital. Future studies looking at promoting increased physical activity among older inpatients should aim to implement interventions particularly during these sedentary periods.

We also used an activity cut-point of four steps or more per minute and found that participants achieved this for 40 min on average per day. This is consistent with findings from other studies, which have found inpatients to be in an upright position for about 43 min [10] and 48 min [28] per day. Interestingly, we did not see the same peaks of walking activity using the cut-point (Fig. 2) as we did from the median step count (Fig. 1a). A possible explanation for this discrepancy is that a more mobile subgroup within our sample undertook most of the walking activity, with the extra steps in each of their active minutes (above the four steps needed to meet the cut-point) then not being counted. This idea is supported by the positively skewed distribution of the daily step count (as shown in Table 1); for example, the most active individual had a daily count of around 3500 steps.

We saw very low mean daily acceleration at the wrist(8.8 milli-g), with 10.9 milli-g previously suggested as a cut-off for sedentary behaviour for community-dwelling individuals [29]. There was little variation in the mean level of acceleration per hour during the daytime (Fig. 1b). By contrast, there appeared to be a meaningful variation in the number of minutes spent at or above our 12 milli-g cut-point, presumably related to upper limb activity during personal care and mealtimes (Fig. 2). These findings would suggest that the assessment of wrist acceleration in this population benefits from minute-by-minute analysis with a low threshold for activity.

While the acceptability for both devices were high (GENEActiv: 96%; SAM 83%), the wrist-worn GENEActiv was found to be more acceptable to patients than the ankle-worn SAM. This is consistent with previous reports of higher compliance of wrist-worn devices [30]. The main negative feedback received regarding the SAM was that it was uncomfortable to wear during sleep time. For future studies, an alternative approach would be to allow patients who find the SAM uncomfortable during sleep time to remove it at night and to put it on again in the morning. The caveat to this would be that night time ambulation may be missed and it would also depend on participants remembering to put it back on in the morning, both of which may affect the accuracy of the data collected. Additionally, difficulties may arise in standardising and enforcing the protocol for daily removal and re-siting of the monitor.

The benefit of using the GENEActiv as shown in this study is the ability to capture upper limb activity, which is important in the rehabilitation process of older inpatients. It also appears to be more acceptable to patients than the ankle-worn device. However, previous studies have shown that wrist-worn devices are less accurate in measuring lower limb and walking activity [11]. The strength of the SAM is in its accuracy in step counting.

### Strengths and imitations

We have undertaken a comprehensive assessment of physical activity in a sample of older medical inpatients using devices worn for several days on both the ankle and the wrist. We have used detailed minute-by-minute analyses of the physical activity data, allowing us to detect the short bouts of activity which appear typical in this population.

Given the smaller sample size of this study and the inclusion criteria that requires participants to provide valid written consent, our findings may not be generalizable to all acute older medical inpatients. The participants recruited are likely to be functionally and cognitively more robust than older medical inpatients in general, who are likely to have lower physical activity levels than we measured.

## Conclusions

Physical activity levels among 38 acute medical inpatients aged ≥ 70 years were very low, with most activity occurring in bouts of less than 5 min duration. Accelerometers can provide useful information to improve our understanding on patients’ activity levels in hospital. A wrist-worn device may provide valuable extra information in older inpatients, where time spent walking is particularly low. Periods of low activity levels such as during the afternoon were identified in this study, which could offer an opportunity to clinicians and researchers interested in promoting increased inpatient physical activity to prevent deconditioning.
